# Anticryptosporidium Efficacy of *Olea europaea* and *Ficus carica* Leaves Extract in Immunocompromised Mice Associated with Biochemical Characters and Antioxidative System

**DOI:** 10.3390/cells10092419

**Published:** 2021-09-14

**Authors:** Wafaa Fayez Abd El-Hamed, Nahed Samy Yousef, Yasser S. A. Mazrou, Walaa A. E. S. Elkholy, Amal I. El-Refaiy, Faten A. Elfeky, Muayad Albadrani, Ahmed I. El-Tokhy, Khaled Abdelaal

**Affiliations:** 1 Biological and Environmental Sciences Department, Faculty of Home Economics, Al-Azhar University, Tanta 31732, Egypt; memo_modern2010@yahoo.com (W.F.A.E.-H.); elrefaiyamal@gmail.com (A.I.E.-R.); 2 Food Science and Technology Department, Faculty of Home Economics, Al-Azhar University, Tanta 31732, Egypt; nahedsamy137@yahoo.com; 3 Business Administration Department, Community College, King Khalid University, Guraiger, Abha 62529, Saudi Arabia; ymazrou@kku.edu.sa; 4 Faculty of Agriculture, Tanta University, Tanta 31512, Egypt; 5 Department of Parasitology, Faculty of Medicine Al-Azhar University, Tanta 11754, Egypt; walaaelkholy76@gmail.com; 6 Zoology Department, Faculty of Science (Girls), Al-Azhar University, Tanta 11754, Egypt; fatenelfeky93@gmail.com; 7 Department of Family and Community Medicine, College of Medicine, Taibah University, Madinah 41541, Saudi Arabia; muayads1@yahoo.com; 8 Plant Protection Department, Faculty of Agriculture, New Valley University, El-Kharga 72511, Egypt; a.tokh@agr.nvu.edu.eg; 9 Excellence Center (EPCRS), Plant Pathology and Biotechnology Laboratory, Faculty of Agriculture, Kafrelsheikh University, Kafr Elsheikh 33516, Egypt

**Keywords:** cryptosporidiosis, enzymes activity, oocyst, nitazoxanide, fig and olive leave extracts, oxidative stress

## Abstract

Cryptosporidiosis is caused by an opportunistic protozoan parasite (*Cryptosporidium parvum* and *C. hominis*) known as a parasite of humans, especially children and immunocompromised patients. The current study was designed to evaluate the therapeutic efficacy of a mixture of fig and olive leaf extracts as an alternative medicinal plant. Parasitological examination for oocysts in the stool and histopathological alterations in the small intestines were examined. Additionally, biochemical analyses of liver and kidney functions in addition to antioxidant parameters such as superoxide dismutase (SOD), glutathione peroxidase (GSH) and catalase (CAT) in the plasma were evaluated. Our results showed that marked reduction in oocysts shedding and amelioration in intestinal histopathological changes and hepatic or renal functions were detected in all treated groups compared to the control infected group. Additionally, the treated groups with tested extracts at ratios 1:3 and 1:5 showed a significant decrease in the number of oocysts compared to the other treated groups. Results exhibited a significant increase in the plasma SOD, CAT and GSH levels in treated groups compared to the infected control one. This study suggested that a mixture of fig and olive leaf extracts is a convenient promising therapeutic agent for Cryptosporidiosis.

## 1. Introduction

Cryptosporidiosis is an opportunistic globally distributed parasitic disease caused by protozoan *Cryptosporidium* [1]. The apicomplexan parasite *Cryptosporidium* infects the intestinal epithelium [2]. Gastrointestinal inflammation occurring via the intestinal parasites is frequently accompanied by functional disturbances, marked modifications in the structure and chemical content of the gut. The body’s antioxidant defenses include scavenging enzymes such as superoxide dismutase (SOD), glutathione peroxidase (GSH) and catalase (CAT) [3]. CAT activity is adversely affected by oxidative stress caused by various diseases such as toxoplasmosis [4].

Organs and tissues with high metabolic and energy requirements have oxidative stress [5]. Oxidative stress leads to cell damage and tissue injury due to an imbalance of oxidants and antioxidants. Oxidative stress has been reported in a rat model induced by *C. parvum* infection to induce tissue damage [6,7]. Oxidative stress has an important role in inducing the occurrence and development of most diseases in both animal and human studies. Furthermore, antioxidants have important protective effects against nearly 50 pathogenic diseases [8]. In acute infection, oxidative stress and free radicals production occur as a result of glucose metabolization for energy production and parasite growth. However, the immune system is stimulated, resulting in defense mechanisms that employ antioxidant enzymes as SOD and GSH to limit free radicals production [9]. Superoxide dismutase (SOD) acts as a first line of defense, as it prevents the formation of new radicals and converts the existing molecules into less harmful ones. This occurs through dismutation of superoxide radicals into hydrogen peroxide (H_2_O_2_) and oxygen that participates in neutralization and depletion of toxic free radicals. Glutathione (GSH) plays an important role in counteracting oxidative damage and cell death [10,11]. These antioxidant enzymes play an important role in scavenging reactive oxygen species such as super oxide and hydrogen peroxide and protect the cell against oxidative stress and, finally, cell death [12,13,14,15,16].

Obiad et al. [17] found no significant differences between the GPT, GOT and urea levels in both mice infected with cryptosporidium and treated with *Curcuma longa* rhizomes, *Coriandrum sativum* and *Viscum album* fruits compared with infected control mice. El Mahalawy et al. [18] showed a significant increase in GOT and GPT levels in an infected group compared to healthy mice. Additionally, Hafez and El Hamed [1] observed an improvement of liver enzymes levels (GOT, GPT) in treated groups compared to the infected control group.

Currently, there is no vaccine, and Nitazoxanide (NTZ) is the standard drug for cryptosporidiosis [19]. Previous studies have found that many plants such as *Euphorbia hirta*, *Ficus carica* and *Ficus religiosa* exerted antiparasitic, antiprotozoal, molluscicidal and insecticidal effects [20]. Most cultures around the world have extensive knowledge of herbal medicinal products. Therapy and preventive plants were used by 75 percent of the world’s population [21]. Due to the great need to develop new anti-cryptosporidial agents, many trials were designed to test the potency of traditional medicinal plants for treating cryptosporidiosis [22]. The mixture of methanolic extracts from fig leaves and olives was the most effective and induced antimicrobial activity against some strains of foodborne disease-causing bacteria and spoilage fungi. Fig and olive leaf extracts with proven potential effectiveness can be used as a natural alternative preventative material for controlling food poisoning diseases, preserving nutrients and avoiding health risks in applications of antimicrobial chemical agents [23]. Fig (*Ficus carica*) and olive (*Olea europaea*) leaf extracts have well-known anti-inflammatory and antioxidant effects and are still under investigation for their antiparasitic activity [24]. Fig and olive leaf extracts have strong antioxidant activity to remove free radicals with an optimal concentration of phenols and flavonoids [25]. Therefore, the present study aimed to evaluate the mixture of fig and olive leaf extracts as an anti-cryptosporidial treatment in experimentally infected immunosuppressed mice in comparison with NTZ.

## 2. Materials and Methods

### 2.1. Preparation of Fig and Olive Leave Extracts

Fig leaves (*Ficus carica*) and olive leaves (*Olea europaea*) were collected from a private farm, Ahmed Orabi Association, Kaliobeya, Egypt. Plant leaves were washed with tap water, then with distilled water to remove impurities such as dust, and kept between folds of filter paper to remove excess water from the external surface. Leaves of fig and olive were dried separately under shade, and milled using the laboratory mill. Fifty grams from each milled leaves sample were extracted with 250 mL methanol 70% by stirring for 2 h on magnetic stirrer at room temperature (25 + 2 °C). The extracts were filtered through folded filter paper into a 500 mL round bottom flask and reduced to dryness on a rotary evaporator at 40 ℃water bath temperature. Stock solutions from each plant were prepared using 25 mg of dried extract residue in one mL sterile distilled water [26]. The tested extract consists of mixing of both stock solution of fig and olive leave extracts with ratios as follows 1:1, 1:3, 1:5 and 1:7 [23]. For treated animal, 200 mg of tested extract/Kg body weight was used.

### 2.2. Oocysts Preparation and Isolation

*Cryptosporidium parvum* oocysts were collected from naturally infected calves. Oocysts were concentrated by floatation in Sheather’s sugar solution [27] then identified by Ziehl–Neelsen staining method [28]. Oocysts were washed three times in PBS and counted using haemocytometer [27], then diluted in distilled water to obtain 10^5^ oocysts/mL and used for infection for two days [29].

### 2.3. The Animals

This study was carried out on laboratory-bred Swiss albino male mice (*n* = 49) aged 8 weeks, weighing 17–20 g. They were all free from any parasitic infection on three consecutive days, as determined by examining their stools using the formol—ether concentration method [30] and modified Ziehl–Neelsen technique [28].

### 2.4. Immunosuppression

Immunosuppression was induced by giving the animals synthetic corticosteroids (dexamethasone) orally at a dose of 0.25 mg/g/day for 14 successive days [29].

### 2.5. The Infection

After immunosuppression, all mice in the studied groups except normal control were infected orally with the prepared oocyst inoculum [29].

### 2.6. Experimental Design

**Figure 1:**
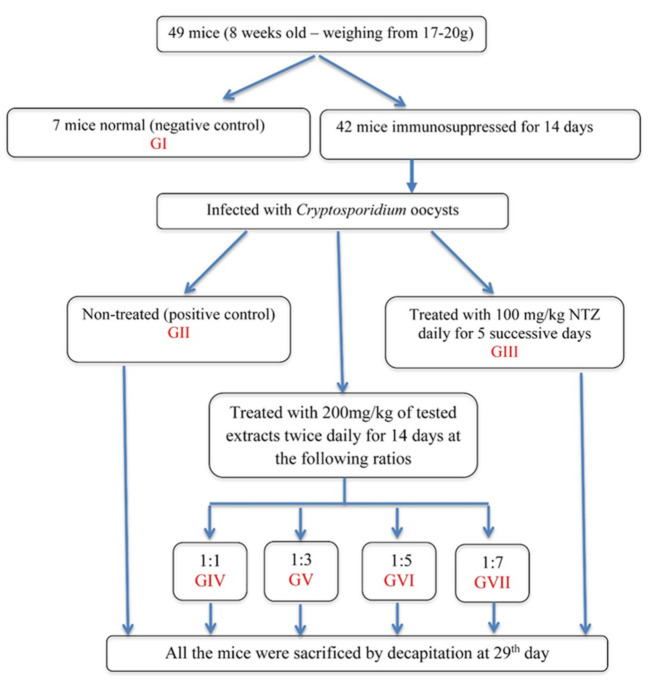
Flow chart of experimental design.

The experimentally forty-nine male Swiss mice were divided equally into seven groups (7 mice/cage), where they were categorized into the following groups; 1: non-immunosuppressed, non-infected and non-treated (negative control); II: immunosuppressed, infected and non-treated (positive control); III: immunosuppressed, infected, treated with NTZ; IV: immunosuppressed, infected, treated with tested extract at ratio 1:1; V: immunosuppressed, infected, treated with tested extract at ratio 1:3; VI: immunosuppressed, infected, treated with tested extract at ratio 1:5; VII: immunosuppressed, infected, treated with tested extract at ratio 1:7 (Figure 1).

The infected mice were treated orally by using a tuberculin syringe connected to a polythene tube [29] either with NTZ at a dose of 100 mg/kg daily starting on day 15 PI for five successive days (group III) [31,32] or with 200 mg/kg of tested extracts twice daily (groups IV to VII) for 14 successive days. The drug and tested extracts was administrated to the mice using the same special syringes that were used for the oocyst inoculation [31,32].

### 2.7. Fecal Samples

Fresh fecal pellets were collected from each group of mice at the end of experiment to counting oocysts of *Cryptosporidium* sp. In 1 mL of specimen sediments, 50 μL from the sediment was taken, and then fecal smears were stained with a modified Ziehl–Neelsen stain. The mean of three counts of oocysts was calculated per high-power field and multiplied by 20 to get the number of oocysts in 1 mL [33].

$$\mathrm{percent}\;\mathrm{of}\;\mathrm{resistance}\;\mathrm{to}\;\mathrm{infection}=\frac{\mathrm{Mean}\;\mathrm{number}\;\mathrm{of}\;\mathrm{oocysts}\;\mathrm{in}\;\mathrm{control}\;\left(\mathrm{GII}\right)-\mathrm{mean}\;\mathrm{oocysts}\;\mathrm{in}\;\mathrm{test}\;\mathrm{group}}{\mathrm{Mean}\;\mathrm{number}\;\mathrm{of}\;\mathrm{oocysts}\;\mathrm{in}\;\mathrm{the}\;\mathrm{control}\;\left(\mathrm{GII}\right)}\times 100$$

### 2.8. Measurement of Serum Biochemical Parameters

At the end of the experiment, all the mice were sacrificed by decapitation. Blood was collected in sterile tubes and left to clot at 4 ℃, then centrifuged for 10 min at 3000 rpm. The serum was genteelly separated from clotted blood (17). For measuring the aspartate, aminotransferase (AST) and alanine aminotransferase (ALT) levels in serum were measured for the liver [34], while levels of urea in serum were measured photometrically [35]. No significant difference in weights of animals of GII: immunosuppressed, infected and non-treated (positive control) or other-treated groups (GIII–GVII) after 14 days (for dose) and after 29 days compared to normal group.

### 2.9. Evaluation of Oxidative Stress and Antioxidant Markers

Different oxidative stress-related biochemical parameters (SOD, CAT and GSH) in plasma were estimated. Superoxide dismutase (SOD) and catalase enzyme activity were estimated [36]. GSH was determined by estimating free—SH groups, USING 5–50 dithiobios 2-nitrobenzoic acid (DTNB) [37].

### 2.10. Histopathological Examinations

Ileal tissues from mice of each group were rapidly removed, fixed in 10% formol saline and submitted to the Pathology Department, Faculty of Medicine, Al-Azhar University, for routine processing. Serial sections 5 μm in thickness were stained with hematoxylin and eosin (H and E) for detection of histopathological alterations and *Cryptosporidium* developmental forms [38].

### 2.11. Statistical Analysis

All results were presented as mean ± standard error (SE). Results were analyzed statistically by one-way analysis of variance (ANOVA) using SPSS 15.0 program, IBM Company (NY, USA) and a value of *p* < 0.05 was considered statistically significant.

## 3. Results

### 3.1. Parasitological Results

**Figure 2:**
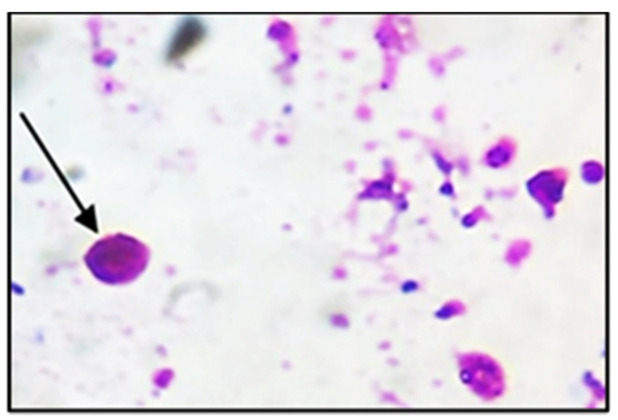
*Cryptosporidium* oocysts in the stool Modified Ziehl–Neelsen stain (MZN stain X1000). The black arrow referred to *Cryptosporidium* oocysts.

The shedding oocyst in the stool was observed with Modified Ziehl–Neelsen stain (MZN) stain as spherical pink organisms (Figure 2). NTZ and other treated groups revealed high significant reduction in oocyst numbers (*p* < 0.0001) compared to the infected control group II. Table 1 shows that all mice groups treated with tested extracts (IV-VII) showed a high significant decrease in the oocyst numbers than that of the drug-treated group III (*p* < 0.001).

Tested extract-treated groups showed 75% reduction in shedding oocyst in the group IV with no significant difference compared to NTZ-treated group. Groups V, VI and VII showed 80%, 84% and 72 % reduction, and with significant difference *p* < 0.0001 compared to control group (II) (Table 1).

### 3.2. Biochemical Results

Our results in Table 2 reveal that the mean ± SE of serum ALT, AST and urea levels were 124 ± 5.0, 226.9 ± 2.8 and 52.7 ± 3.6, respectively, in the control infected group II with high significant increase (*p* < 0.001) compared to the normal control group I (86 ± 3.5, 176.6 ± 16.5 and 37.3 ± 0.9, respectively). The drug-treated group (GIII) showed high significant changes in ALT levels (*p* < 0.0001) compared to both normal and infected control groups (GI and GII) with a significant (*p* < 0.05) decrease in AST level compared to the infected control group (GII) and high significant increase (*p* < 0.001) in blood urea levels compared to the normal control (GI). All treated groups (GIV–GVII) showed no significant changes in ALT, AST or urea compared to the normal control group I and relatively significant changes with both infected control (GII) and drug-treated (GIII).

### 3.3. Histopathological Changes

**Figure 3:**
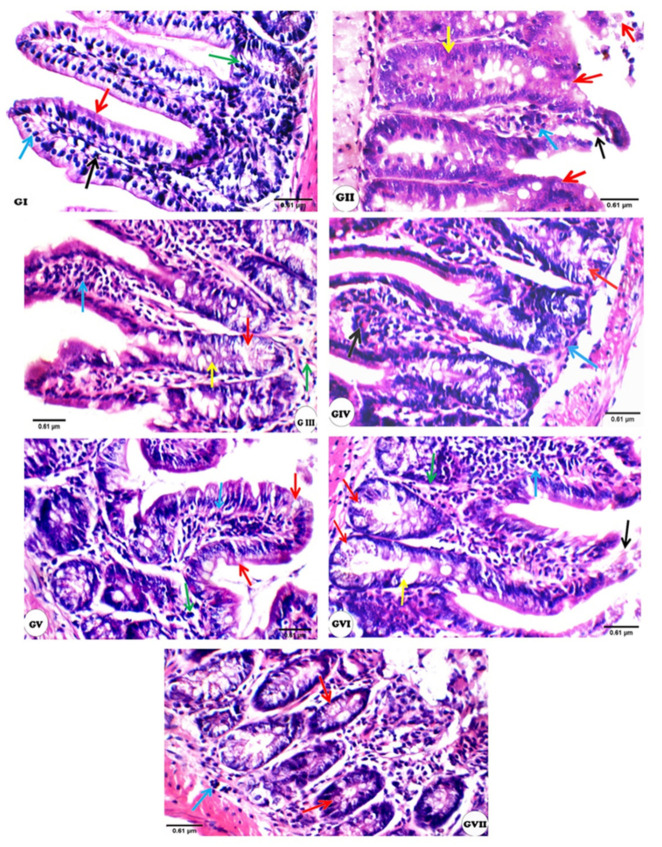
The changes in the intestinal sections of different experimental groups (H&E at high power view × 400): GI; (Negative control): showing average villi/crypt ratio and villi composed of connective tissue core (black arrow) covered by tall columnar epithelial cells (blue arrow) with intact brush border (red arrow), and average crypts (green arrow), GII (Positive control); showing very short and thin villi with loss of epithelial covering (black arrow), excess oocysts in superficial mucosa (red arrow) with mild interstitial (blue arrow) and submucosal inflammatory infiltrate with scattered eosinophils (green arrow), and markedly elongated crypts (yellow arrow), GIII (Drug-treated); showing relatively short villi (black arrow), scattered oocysts in the crypts (red arrows) with mild intra-villous (blue arrow) and submucosal inflammatory infiltrate with scattered eosinophils (green arrow), and relatively elongated crypts (yellow arrow), GIV (tested extract ratio 1:1); showing broad villi (black arrow), scattered oocysts in the crypts (red arrows) with mild intra-villous inflammatory infiltrate (blue arrow), GV (tested extract ratio 1:3); showing relatively short broad villi (black arrow), scattered oocysts in superficial mucosa (red arrows) with mild intra-villous (blue arrow) and interstitial inflammatory infiltrate (green arrow), GVI (tested extract ratio 1:5); showing relatively short broad villi with partially necrotic tip (black arrow), excess oocysts in the crypts (red arrow) with marked intra-villous (blue arrow) and interstitial inflammatory infiltrate (green arrow), and relatively elongated crypts (yellow arrow), GVII (tested extract ratio 1:7); showing scattered oocysts in the crypts (red arrows) with marked interstitial (black arrow) and mild submucosal inflammatory infiltrate with scattered eosinophils (blue arrow).

Histopathological changes in H&E stained intestinal sections of different groups were shown in Figure 3 and Table 3. The intestinal wall of the normal control group showed an average mucosal thickness with average villi composed of connective tissue core covered by tall columnar epithelial cells with intact brush border. An average crypts, villi/crypt ratio and musculosa can also be detected (Figure 3GI). The intestinal wall of the infected control group showed marked reduction of mucosal thickness, and short blunted and fragmented villi with loss of epithelial covering. Excess oocysts in superficial mucosa with mild interstitial and submucosal inflammatory infiltrate with scattered eosinophils and markedly elongated crypts could be detected (Figure 3GII). Regarding the drug-treated group III, Mild scattered oocysts and inflammatory cellular infiltrates with an average mucosa and relatively thin or broad villi were detected (Figure 3GIII). Other treated groups with tested extract (GIV–GVII) revealed an average mucosal thickness with relatively long or short thin villi, relatively elongated crypts as well as an average submucosa. However, mild oocysts were detected in groups IV, V and VII.

### 3.4. Oxidative Stress Parameters in the Plasma of Mice Infected with Cryptosporidium parvum

Cryptosporidiosis affects antioxidant enzymes activity in the blood plasma of mice groups as revealed in Table 4, showing a significant reduction (*p* < 0.0001) in GSH, SOD and CAT in infected control group (GII) compared to the normal control (GI). On the other hand, the NTZ-treated group as well as groups treated with testes extract at different ratios significantly corrected their levels toward the normal control levels. GSH in groups VI and VII, SOD and CAT in group V reported more amelioration of antioxidant activities than other treated groups with tested extract.

## 4. Discussion

*Cryptosporidium* infection has been proposed to be one of the major causes of diarrhea in humans, which is a self-limited illness lasting between two and four weeks, but can be life-threatening in immunocompromised patients [39]. Based on the recommendations of WHO to use products of some medicinal plants to replace synthetic ones in current medicine, trials were designed to test the potency of some plants for treating cryptosporidiosis [40]. Both olive leaf extract and fig leaf extract have anti-inflammatory and antioxidant properties. There is no published research on the effects of a mixture of fig and olive leaf extracts with different ratios for treating parasites, but this mixture of fig and olive leaf extracts with different ratios used as a strong anti-microbial effect [23]. In the present study, an assessment of the effect of the fig and olive leaf extract mixtures at different ratios on cryptosporidiosis mice was done. Parasitological results revealed a high significant decrease in the number of oocysts in all groups treated with the mixture of fig and olive leaf extracts at different ratios revealed a high significant decrease compared to both infected control as well as NTZ-treated group. The previous study reported that there was a 100% decrease in the secretion of *Cryptosporidium* oocyst in the stool of the infected mice treated with *Olea europaea* two weeks after administration. Thus, *O. europaea* may be used as a natural remedy for cryptosporidiosis. The anti-parasitic effect of olive pomace is due to the presence of oleuropein, which is the main component of *O. europaea* leaves [41]. Group VI in this study revealed a high significant decrease in the oocyst numbers 84% than that in the drug-treated group III. This finding is comparable to numerous previous results from studies for treating the parasite with other plant extracts, each of which had some impact, but none of which were curative [42]. The effect of *Punica granitium* was 53% at 1000 mg/kg, while *Thymus vulgaris* was 50% at 1000 mg/kg [39].

Egyptian propolis extracts have an average effect on cryptosporidiosis in dexamethasone-immunosuppressed rat model as evidenced by decreased oocysts shedding [43]. In the current study, the serum ALT, AST and urea have been corrected toward normal control at the different concentrations of tested extracts. Serum levels of liver enzymes (AST and ALT) and serum urea showed high significant increase in the infected control group compared to the normal control one. This is consistent with earlier studies that found an increase in liver enzymes during *Cryptosporium* infection [1,18,44]. This can be explained due to hepatocytes damages that induced by the parasite as elevated AST indicates mitochondrial damage and elevated ALT signaling cell membrane injury [45]. The levels of these biochemical parameters were significantly restored in all treated groups towards normal level with more improvement in the Fig and olive leave extracts-treated groups compared to NTZ-treated one. This can be referred to the suppressive effect of the mixture against the infection with minimizing hepatocytes damage. In addition, groups treated with the mixture at ratio 1:3 and 1:5 revealed more amelioration in the liver enzymes.

Regarding histopathological changes, marked alterations such as short blunted and fragmented villi with loss of epithelial covering, as well as excess oocysts were detected in the control infected group. This result should be attributed to the immunodeficient situation that flared up the infection. This was evident research [46], and another study [47] found a higher number of developmental stages in the epithelial cells of the infected control group’s gut [47,48]. A reduction in the villus to crypt ratio in mice infected with *C. parvum* oocysts was observed by some studies. In addition, the shortening and fusion of villi, numerous oocysts adhering to the intestinal mucosa in Rats infected with *C. parvum* oocysts orally 4 × 10^5^ and received dexamethasone for 7 successive days was shown [2,7]. An amelioration of all histopathological changes with decreased number of oocysts was detected in tested extracts, and nitazoxanide-treated groups successfully decreased *Cryptosporidium* oocysts and relative regaining of the intestinal architecture, while the GV (1:3) and GVI (1:5) intestinal walls were more correct toward the normal group [49], indicating that plant oils might compete for block receptor sites on the surface of the ileum, thus leading to reduction in *C. parvum* colonization. The balance of living organisms must be maintained by the stability between oxidative and antioxidant defense [50]. In this study, a significant decrease in the antioxidant parameters that included GSH, SOD, and CAT was observed in the plasma of the positive control group (GII) compared to the negative control group (GI). Infected immunocompromised mice had lower levels of SOD, GSH, and CAT in their livers and intestines, suggesting that free radical-induced oxidative stress played an important role in the development of *C. parvum* infection in mice [3,7]. Loss of body condition, profuse bloody diarrhea, and peak oocyst shedding severity occurred at the 10th day DPI. The reduction of antioxidant enzyme activities, such as SOD, GST and CAT, might lead to a significant depletion in levels of hepatic GSH. As a result, decreased GSH levels in restraint stressed rats may be related to increased plasma membrane susceptibility to peroxide outbreak [51,52].

In the current study, animals treated with mixture of fig and olive leaf extracts in different ratios observed a significant increase in levels of GSH, SOD and CAT toward the normal group (GI). It may be due to oleuropein in olive and psoralen in Figure Oleuropein is a biophenol with many biological activities. Oleuropein has been shown to have leishmanicidal effects in vitro against three *Leishmania* spp. and to reduce parasite burden in *L. donovani*-infected Balb/c mice [53]. Amotosalen, a psoralen drug, has been used to minimize the pathological effects of *L. major* inoculation as well as the virulence of *L. infantum* and *L. chagasi* promastigotes [54,55]. A previous study found that fig fruits and olive leaf extracts reduced oxidative stress (LPO, NO) and improved skin lesions, promoting healing and parasite resolution in mice infected with *Leishmania* major, with full healing after 21 days when compared to standard drug Pentostam. The presence of phenolic compounds may be responsible for this behavior [56].

Rats treated with garlic extract showed a significant increase in GSH, CAT, and SOD [57]. The same results observed when treated cryptosporidiosis Swiss albino mice with S-Methylcysteine at doses of 25 and 50 mg/kg body weight. significantly led to correcting the levels of GSH and as SOD activity toward the control level [18]. The antioxidant potential of crude extract of *Solanum nigrum* leaves and its active constituents as treatment against restraint stress in rat’s liver was evaluated, and it was found that tested extract was confirmed by the deterioration of stress change in ALT and AST marker enzymes toward their normal values [58]. The anti-*Cryptosporidium* effect may be caused by the presence of many individual phytochemical molecules or a synergy in-between. A Tunisian experiment reported that the olive leaf extract of five different Tunisian olive varieties had antioxidant and leishmanicidal activities and relates that effect to the flavonoids present in the extract, which are broad classes of plant phenolics, which are known to possess a well-established protective effect against membrane lipoperoxidative damages with potent antiparasitic activity [59]. *Ficus carica* and *Olea europaea* have strong antioxidant potency to scavenge free radicals at an optimal phenolic and flavonoid concentration [22].

## 5. Conclusions

Based on our findings, it can be concluded that a combination of fig and olive leaf methanolic extracts has good efficacy and is a promising therapeutic agent against *Cryptosporidium*, diminishing the oocysts shedding, protecting the intestinal epithelial from deleterious effects of *C. parvum*, and significantly increasing in levels of GSH, SOD and CAT toward the normal group. The plant’s traditional usage in parasitic illnesses was validated by all treated groups. Further investigation is recommended on the applications of the fig mixture and olive leaf extract as a complementary medicine in treating other parasitic infections to reduce chemical drugs application and safe human health.

## Figures and Tables

**Table 1:** The number of *Cryptosporidium* shed oocysts in different experimental groups.

Experimental Groups	Oocysts Number	Resistance to Infection (%)
II	± 203.7 ^+++^	−
III	5362 ± 23.8 ***	56.4
IV	3000 ± 10.8 ***^,+++^	75.6
V	2476 ± 30.4 ***^,+++^	79.9
VI	1995 ± 6.5 ***^,+++^	83.8
VII	3434 ± 22.9 ***^,+++^	72.0

All data are expressed as mean ± standard errors. *p* < 0.05 *: significant difference compared to infected control group (II), ^+^: significant difference compared to drug-treated group (III), ***,^+++^: *p* < 0.0001.

**Table 2:** Serum biochemical parameters (ALT, AST and urea) in experimental groups.

Experimental Groups	ALT	AST	Urea
I	86 ± 3.5 ^+++,###^	176.6 ± 16.5 ^+++^	37.3 ± 0.9 ^+++,###^
II	124 ± 5 ***^,###^	226.9 ± 2.8 ***^,#^	52.7 ± 3.6 ***
III	198.6 ± 11.4 ***^,+++^	198.6 ± 11.8 ^+^	57.2 ± 2.8 ***
IV	92.5 ± 6.6 ^++,###^	183 ± 2.4 ^++^	39 ± 1 ^+++,###^
V	89 ± 6.2 ^+++,###^	172.5 ± 5.4 ^+++,#^	37 ± 2.4 ^+++,###^
VI	88 ± 2.4 ^+++,###^	174.5 ± 1.2 ^+++,#^	38 ± 1.4 ^+++,###^
VII	88.7 ± 2.4 ^+++,###^	178.4 ± 3.6 ^+++^	38.7 ± 2.4 ^+++,###^

All data are expressed as mean ± standard errors. *p* < 0.05 *: significant difference compared to normal control group (I). +: significant different compared to infected control group (II). ^#^: significant control compared to drug-treated group (III). ^+^, ^#^: *p* < 0.05; ^++^, *p* < 0.001; ***, ^+++^, ^###^: *p* < 0.0001.

**Table 3:** Histopathological changes in the intestinal sections of different groups.

Experimental Groups	Mucosal Thickness	Villi	Crypts	Inflammatory Infiltrate	Oocysts	Submucosa	Musculosa
I	0	0	0	0	0	0	0
II	++	++	++	++	++	++	0
III	0	+	+	+	+	+	0
IV	0	+	+	+	+	0	0
V	+	++	+	++	+	++	0
VI	0	+	+	++	++	0	0
VII	+	++	+	++	+	+	0

Mucosal thickness: 0: Average; +: Relatively thin; ++: Marked reduction. Villi: 0: Average; +: Short/thin/broad; ++: Markedly fragmented/necrotic. Crypts: 0: Average; +: Relatively elongated; ++: Markedly elongated. Inflammatory infiltrate: 0: No; +: Mild; ++: Moderate/marked. Oocysts: 0: No; +: Scattered; ++: Excess. Submucosa: 0: Average; +: Mild inflammatory infiltrate; ++: Marked inflammatory infiltrate. Musculosa: 0: Average; +: Mild inflammatory infiltrate; ++: Marked inflammatory infiltrate.

**Table 4:** Antioxidant enzymes activity in plasma of mice groups.

Experimental Groups	Oxidative Stress Parameters		
	GSH, U/L	SOD, U/mL	CAT, U/mL
I	7.6 ± 0.08 ^+++,###^	273.4 ± 3.3 ^+++,###^	88.8 ± 3.2 ^+++,###^
II	1.01 ± 0.07 ***^,###^	72 ± 2.5 ***^,###^	52.6 ± 0.9 ***^,###^
III	2.7 ± 0.03 ***^,+++^	218.6 ± 1.03 ***^,+++^	113.8 ± 1.8 ***^,+++^
IV	3.5 ± 0.04 ***^,+++,###^	214.2 ± 3.2 ***^,+++^	78.2 ± 2.03 ***^,+++,###^
V	3.4 ± 0.03 ***^,+++,###^	227.2 ± 1.9 ***^,+++,#^	88.4 ± 1.5 ^+++,###^
VI	3.6 ± 0.04 ***^,+++,###^	250.6 ± 3.1 ***^,+++,###^	75.2 ± 1.7 ***^,+++,###^
VII	3.8 ± 0.07 ***^,+++,###^	287.4 ± 4.5 ***^,+++,###^	78.2 ± 3.5 ***^,+++,###^

All data are expressed as mean ± standard errors. *p* < 0.05. *: significant difference compared to normal control group (I). ^+^: significant different compared to infected control group (II). ^#^: significant control compared to drug-treated group (III). ^#^: *p* < 0.05; ***, ^+++^, ^###^: *p* < 0.0001.

## Data Availability

Data Availability Statements in section “MDPI Research Data Policies” at https://www.mdpi.com/ethics (accessed on 12 August 2021).
